# Factors associated with suicidal ideation in drug addicts based on the theory of planned behavior

**DOI:** 10.1186/s12888-021-03387-9

**Published:** 2021-07-26

**Authors:** Ali Khani Jeihooni, Mehdi Amirkhani, Tayebeh Rakhshani, Pooyan Afzali Hasirini, Hanieh Jormand

**Affiliations:** 1 Nutrition Research Center, Department of Public Health, School of Health, Shiraz University of Medical Sciences, Shiraz, Iran; 2 Department of Nursing, School of Nursing, Fasa University of Medical Sciences, Fasa, Iran; 3 Department of Public Health, School of Health, Kermanshah University of Medical Sciences, Kermanshah, Iran; 4 Department of Health Education and Promotion, School of Health, Hamadan University of Medical Sciences, Hamadan, Iran

**Keywords:** Suicidal ideation, Drug addicts, Theory of planned behavior

## Abstract

**Background:**

Several studies have attempted to understand the link among drug addicts and suicidal ideation. The purpose of this study was to investigate the factors associated with suicidal ideation in drug addicts based on the Theory of Planned Behavior (TPB).

**Method:**

This cross-sectional study was conducted with 2160 of drug addicts in private and public clinics for quitting addiction through methadone treatment in Shiraz city, Fars province, Iran from October 2018 to June 2019. Data gathering tools were a questionnaire on demographic characteristics, the Beck Scale for Suicidal Ideation and a questionnaire based on constructs of TPB. Data were analysed by SPSS 22 using descriptive statistics (mean, standard deviation, and frequency) and statistical tests (independent t-test, Pearson correlation coefficient, and linear regression). The significance level was considered 0.05.

**Results:**

The mean age of the participants was 39.24 ± 11.92; 80.28% of them had a history of quit and 43.19% of them had a history of arrest and imprisonment. According to the results, 19.63% of the participants had suicidal ideation and 10.97% had a history of suicide attempt during their lifetime. The constructs of attitude, subjective norms, perceived behavioral control, and intent predicted suicidal ideation in the subjects. Intent and perceived behavioral control constructs were the strongest predictors of suicidal ideation, respectively. In general, the studied variables predicted 54.8% of suicidal ideation.

**Conclusion:**

The structures of attitude, subjective norms and perceived behavioral control and intent predicted suicidal ideation in the drug addicts, so the theory of planned behavior will be a good framework for educational interventions to reduce suicide in them.

## Introduction

Suicide is one of the most important mental health problems and abnormal social behaviors that leads to personal and family losses as well as social harm. A Suicide attempt is consciously aimed at self-harm and resulting from suicidal ideation. It occurs mostly in introverted, anxious, depressed, and those who are socially unable to communicate [1]. Suicide is the third leading cause of death in the American adolescent population [2]. Various studies indicated that one of the most important predisposing factors for suicide attempt planning is suicidal ideation which ultimately leads to death. Suicidal ideation is assumed to be different geographically and culturally and is influenced by psychological, religious, social, and economic factors. Suicidal ideation ranges from fleeting thoughts such as the futility of life to the preoccupation with self-harm [3].

One of the problems of the international community that has led to severe physical and mental health disorders is drug dependence, which leads to different misbehaviors including suicide attempts [4] so that the most common cause of death in the population of addicts is suicide. And half of those who attempt suicide have a history of drug abuse [5]. In a study conducted between 2011 and 2016 on the suicide rate in southern Iran, the suicide and death rates were 21.47 and 4.52 per 100,000, respectively [6]. According to the World Health Organization, 2.5 million people worldwide die each year from drug addiction. Studies on people who have attempted suicide show that 19–63% have drug-related disorders [7].

**Fig. 1:**
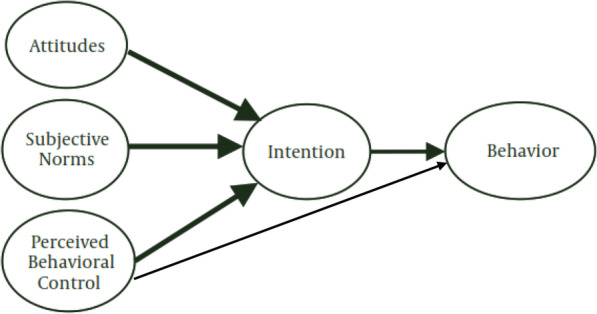
Theory of Planned Behavior [11]

Because most health problems are closely related to human thought and behavior, behavioral theories can be used to understand how to prevent and control health problems. One of the models of behavior change is the theory of planned behavior (TPB). Theory of Planned Behavior (TPB) is one of the most.

frequently used theories and educational models to analyze healthy or unhealthy behaviors. Several pieces of evidence emphasized the effectiveness of the TPB constructs for explaining the high-risk behaviors [8–11]. According to the TPB, the intention to perform a behavior is predicted by three factors: (a) attitude that is a positive or negative evaluation of the individual’s behavior; (b) subjective norms that refer to the social pressure perceived by the individual to behave or not; and (C) perceived behavioral control refers to the degree of the voluntary control feeling of individuals to do or not to do a behavior. According to this construct, when there is no limit for acceptance of a particular behavior, the individual may have complete control over behavior, and vice versa. Because the behavior may require factors such as resources, facilities, skills, etc. [11, 12]. According to which the most important factor determining a person’s behavior is the intention, indicating his motivation to adopt behavior and is influenced by various factors. Also, according to this theory, behavioral intention is the result of attitude towards behavior, which consists of two sub-constructs of behavioral beliefs and evaluation of results of behavior that lead to attitude toward behavior. Individuals usually act on their perceptions of what others think they should do, and their intention to accept behavior is potentially influenced by people with whom they have close relationships Fig. 1 [11, 13].

A lot of research has been conducted toward identifying reasons and factors affecting substance use disorders among adulthood [14, 15]. The development of tolerance and physiological and psychological dependence on addictive substances can lead to irritability, aggression, and other psychological symptoms. In general, this process reduces physical functioning, undermines psychosocial capabilities, and decreases the mental health dimension. However, mental state disorders usually encompass those psychiatric disorders, such as mood, anxiety, and substance use disorders [16]. Thus, personality disorder commonly co-occurs with mental state disorders, causing enormous consequences of substance use disorders [17]. Several studies have shown that mental health problems are associated with an increased risk of mental state disorders [16, 18, 19]. Also, a well-established risk factor for suicidal behavior is the presence of mental disorders, especially mood disorders, substance use disorders, and schizophrenia [20].

But, the reasons and factors affecting the mental state disorders such as Suicide attempts among individuals with substance use disorders have been less studied in the field researches.

Considering the importance of factors associated with suicidal ideation in the population of drug addicts, the need to identify the effective factors in creating a behavior based on the principles of TPB, and to reduce the associated risk factors, it is necessary to identify the most effective variables in creating behavior and their influence on patients’ performance and to design and implement effective educational interventions accordingly. The present study, therefore, aimed to investigate the factors associated with suicidal ideation in drug addicts based on the TPB.

## Materials and methods

This descriptive cross-sectional study was conducted from October 2018 to June 2019. The research population is private and public clinics for quitting addiction through methadone treatment in Shiraz city, Fars province, Iran. Among the private and public clinics in Shiraz, 5 centers (10 in total) were selected by simple random sampling.

According to a study by Shiraly and Kokabi, 88.8, 9, and 2.2% of the general population in Shiraz had a low, moderate, and high risk of suicide, respectively [21]. Also, based on a study by Naghibi et al. 17.9% of the drug addicts referring to Sari addiction treatment centers had suicidal ideation [4], which considering the alpha of 0.05 and accuracy of 0.02, it was calculated equal to 1600 individual. Considering the effect of the population-based design, it was multiplied by a factor of 1.5 and the final sample volume was estimated at 2400 people, of which 2160 people completed the tools used.

Inclusion criteria were male gender and having a case of drug abuse in addiction treatment centers. Exclusion criteria were failure to complete the questionnaire, unsuitable mental and physical condition, and any severe acute diseases which might influence participant’s decision to suicide. This study was approved by the Ethics Committee of Shiraz University of Medical Sciences. After explaining the objectives of the study, written consent was obtained from the participants and they were assured that their information would be treated as strictly confidential. For illiterate people, the text of the consent form was read to them and their fingerprints were recorded. The questionnaire was also completed anonymously. Data were collected by 5 public health experts through a questionnaire in addiction treatment centers.

Data collection tools comprised of 3 parts: 1) demographic characteristics and background information (age, education, occupation, marital status, type of substance used, method of use, age of first use, duration of addiction, history of quit, history of detention and imprisonment, history of addiction in family, and history of suicide attempt); 2) Beck Scale for Suicidal Ideation (BSSI), which measures the severity of suicidal ideation using 19 items (scoring 0–20) [22]. This scale has a good internal consistency and in various studies, its Cronbach’s alpha has been reported 0.84–0.89 [23, 24]. The concurrent validity of suicide risk assessment is reported to be 69% [25].

In this scale, the first 5 questions are posed as screening questions. Questions four and five are the main screening filters in this questionnaire. Question four has three options, and if one chooses either (b) or (c), it indicates a desire to make an active suicide attempt. The options are as follows:

- When I am in a difficult and dangerous situation, I try to save my life.
- When I am in a difficult and dangerous situation, it does not matter to me whether I live or die.
- I do not make any effort to save my life when I am in a difficult situation.

Question five also has three options, and if one chooses either (b) or (c), it indicates a passive suicidal desire. The options are as follows:

- I have no desire to commit suicide.
- Sometimes, I have a slight desire to commit suicide.
- I have a strong desire to commit suicide.

In general, if participants approve of one of the options (b) or (c) of the above two questions, they have an active or passive suicidal desire, and 14 questions are needed to determine the severity of suicidal ideation, otherwise, there is no need to answer other questions [22].

In a study by Anisi et al., the internal correlation of BSSI was reported to be 0.95 and 0.88 based on the Cronbach’s alpha test and the method of halving, respectively. The internal correlation of screening questions was also reported to be 0.88 using Cronbach’s alpha test [26].

The third part of the data collection tools was a questionnaire based on the TPB. The constructs of TPB.

were measured through a designed and validated questionnaire by Rezapur-Shahkolai et al. [3], Aldrich [27], George [28], and Matheson [29]. The questionnaire included 25 three-choice questions evaluating knowledge. The options were (a) *true*, (b) *false*, and (c) *I do not know* (scored 0–25). A five-point Likert scale was used to measure attitude, subjective norms, perceived behavioral control, and behavioral intention. The attitude was measured using 10 questions (with a minimum score of 10 and the maximum score of 50). The subjective norms were measured using 6 questions (with a minimum score of 6 and the maximum score of 30). The perceived behavioral control was measured using 10 questions (with a minimum score of 10 and the maximum score of 50). The behavioral intention was also measured using 5 questions (with a minimum score of 5 and the maximum score of 25).

Data were analyzed by SPSS software using descriptive statistics (mean, standard deviation, and frequency) and statistical tests (independent t-test, Pearson correlation coefficient, and linear regression). The significance level was considered 0.05.

## Results

Out of 2160 patients participating in the study, 19.72 and 80.28% were single and married, respectively. The mean age of the participants was 39.24 ± 11.92; 80.28% of them had a history of quit and 43.19% of them had a history of arrest and imprisonment. According to the results, 19.63% of the participants had suicidal ideation and 10.97% had a history of a suicide attempt during their lifetime. Other demographic characteristics were given in Table 1.

**Table 1:** Demographic characteristics of the participants

Variable		Number	Percentage
Education	Illiterate	24	1.11
	Primary school	724	33.52
	Secondary school	1014	46.94
	High school	356	16.48
	University	42	1.95
Occupation	Employed	86	3.98
	Labourer	375	17.36
	Self-employed	982	45.46
	Retired	183	8.47
	Unemployed	534	24.73
Type of substance used	Cigarette and opium	1116	51.67
	Cigarette and opium residue	404	18.70
	Cigarette and crack	205	9.49
	Cigarette and (heroin, alcohol, etc.)	435	20.14
Method of use	Oral	680	31.48
	Smelling	304	14.07
	Smoking	892	41.30
	Injecting	284	13.15
Age of first use	< 20	890	41.20
	20–30	914	42.31
	30–40	190	8.80
	>40	166	7.69
Duration of addiction	< 5	365	16.90
	5–10	709	32.82
	< 10	1086	50.28
History of addiction in family	Yes	1197	55.42
	No	963	44.58

The mean score of suicidal ideation, knowledge, attitude, subjective norms, perceived behavioral control, and suicide attempt was 7.25 ± 7.72, 9.34 ± 3.13, 28.14 ± 4.76, 13.71 ± 3.44, 26.15 ± 4.82, and 7.25 ± 2.70, respectively.

there was a significant relationship between attitudes, subjective norms, perceived behavioral control, and intent with suicidal ideation (Table 2).

**Table 2:** Correlation between TPB constructs and suicidal ideation

Variables	1	2	3	4	5
Intention	1				
Suicide ideation	0.702	1			
Attitude	−0.508	− 0.564	1		
Subjective norms	−0.414	− 0.558	0.405	1	
Perceived behavioural control	−0.445	−0.478	0.440	0.390	1

The constructs of attitude, subjective norms, perceived behavioral control, and intent predicted suicidal ideation in the subjects. Intent and perceived behavioral control constructs were the strongest predictors of suicidal ideation, respectively. In general, the studied variables predicted 54.8% of suicidal ideation (Table 3).

**Table 3:** Analysis of factors associated with suicidal ideation

Variables	Beta	S.E	B	P	Dependent variation
Intention	−0.224	0.75	−0.115	0.022	Suicidal ideation *R*^2^ = /548 *R*^2^*Adjusted=/169*
Attitude	−0.208	0.82	−0.174	0.037	
Subjective norms	−0.192	0.60	−0.136	0.041	
Perceived behavioural control	−0.215	0.81	−0.122	0.028	

## Discussion

According to the results of this study, 19.63% of drug addicts had suicidal ideation and 10.97% of them had a history of a suicide attempt during their lifetime. Comparison of the results of this study with previous studies showed that the prevalence of suicidal ideation among drug addicts is significant; for example, an international study in 17 countries around the world found that the lifetime prevalence of suicidal ideation is 9.2% [30]. In a study by Malakouti et al., the prevalence of suicidal ideation in the general population was reported 12.7 and 12.8% in women [31]. These differences can be attributed to the geographical, cultural, and social conditions of study populations.

One of the influential variables in predicting suicidal ideation in this study was behavioral control which was to be found as the strongest predictor of suicidal ideation. This variable is one of the important constructs of the theory of planned behavior. Perceived behavioral control is related to an individual’s perception of self-confidence in the ability to perform a behavior. Thus, where individuals believe that a particular behavior is under their control, they are more likely perform the behavior [3]. The results of a study by George showed that perceived behavioral control is a significant predictor of suicidal ideation [28]. Perceived behavioral control, directly and indirectly, may affect the person’s behavior. The predictive power of TPB was reported in many studies on health behaviors [32]. The result of a study by Yadava et al. showed that perceived behavioral control is the most important predictor of suicidal ideation. Also, attitude, subjective norms, and perceived behavioral control explained a total of 72% of the variance of suicidal ideation [33]. The results of a study by Bashirian et al. showed that planned behavior control is a strong predictor of intention to drug abuse [34]. The limited studies conducted on suicidal behaviors based on the TPB generally show that perceived behavioral control is a strong predictor of suicidal ideation [35]. Attitude is another construct of the theory of planned behavior and one of the predisposing factors of suicidal ideation was examined in this study and the final model extracted from linear regression was one of the factors affecting suicidal ideation. Attitudes toward suicide include persistent positive or negative emotions and behavioral avoidance of suicidal behaviors as well as those who commit suicide [36]. A positive attitude towards suicide makes people consider suicide an acceptable issue and therefore increase its attractiveness. A positive attitude in combination with suicidal ideation also increases the likelihood of death in suicidal ideation [37].

Studies show that a person with a high level of suicidal ideation is more likely to have a positive attitude toward suicide, while a person with a negative attitude toward suicide has a lower risk of suicide [38]. In another study, attitudes toward suicide were introduced into the background of suicidal ideation. In this study, the positive attitude toward suicide was reported as an important predictor of suicidal ideation and there was a positive correlation between a positive attitude toward suicide and a high risk of suicide attempt [39]. Attitude is a complete cultural and social issue because the cultural variables of society, different religious beliefs, customs, and traditions affect people’s attitudes towards life and death. It seems that in this study, a positive attitude towards suicide has led to the formation of persistent suicidal ideation. A positive attitude towards suicide can make suicide more attractive and a suitable way to end one’s life in the face of difficulties. In this study, subjective norms also played an important role in predicting suicidal ideation, which was not consistent with the results of some other studies. The results of a study by Khezeli et al. showed that subjective norms have the least role in predicting suicidal ideation [3].

In the study by Bashirian et al. [34], subjective norms were a weaker predictor than other constructs, and other constructs were able to predict 37.2% of the variance of smoking behavior by university students. The results of a study by Seo et al. showed that the subjective norms are not able to predict nutritional intention and behavior that is not consistent with our study. But the results of another study by Babazadeh et al. showed the important role of subjective norms predictor that was consistent with our study [40]. In the study by Seo et al., the intention to consume fast food was found to be strongly associated with subjective norms [41]. Subjective norms are one of the effective factors in performing a behavior. It seems that in drug addicts, social pressure and subjective norms are very effective in forming suicidal ideation.

Also, based on results Intention construct was the strongest predictor of suicidal ideation, respectively. In general, the relationship between behavioral intention and actual behavior especially in high-risk behaviors has been confirmed in several studies [9, 10, 42]. And most of these studies have emphasized the role of behavioral intention as a pre-behavioral stage and a strong risk factor for high-risk behaviors.

It is noteworthy that there is a strong relationship between intention and behavior in this study due to the high average age of participants (39.24 years), which strengthens the hypothesis of rational action, behavior with prior planning and intention without the intervention of social influences in adults, which confirms previous research has conducted behavior in adults [9, 10, 43].

The results of this study showed that the theory of planned behavior is a good predictor for high-risk thoughts and behaviors such as suicide, therefore, it is highly recommended to design and implement effective educational interventions based on the TPB may impact on reduce suicidal ideation in substance use disorders.

### Limitations

One of the limitations of the present study was the limited population of addicts, therefore, it is highly recommended to act cautiously when generalizing the results to other patients or statistical populations. The use of the self-reporting method to collect data was another limitation of the study.

It is suggested to apply the theory of planned behavior to other disorders and high-risk behaviors in other populations, including patients with cancer and other chronic diseases.

## Conclusion

Results of the present study revealed that the constructs of attitude, subjective norms, perceived behavioral control, and intent predicted suicidal ideation in drug addicts, so the theory of planned behavior will be a good framework for educational interventions to reduce suicide in them. we suggest strategies that increase perceptions about the disadvantages of suicide for the individual and the family, enhance the attitude toward help-seeking, treat depression and hopelessness, and reduce the social desirability of suicide among drug addicts. Also considering the role of perceived behavioral control, it is suggested that interventions that enhance life skills such as resilience and anger management be implemented for drug addicts.

## Data Availability

The datasets used and/or analyzed during the current study can be made available by the corresponding author on reasonable request.

## Abbreviations

TPB: Theory of Planned Behavior
BSSI: Beck Scale for Suicidal Ideation
